# PP1C and PP2A are p70S6K Phosphatases Whose Inhibition Ameliorates HLD12-Associated Inhibition of Oligodendroglial Cell Morphological Differentiation

**DOI:** 10.3390/biomedicines8040089

**Published:** 2020-04-16

**Authors:** Naoto Matsumoto, Yuki Miyamoto, Kohei Hattori, Akihiro Ito, Hironori Harada, Hiroaki Oizumi, Katsuya Ohbuchi, Kazushige Mizoguchi, Junji Yamauchi

**Affiliations:** 1 Laboratory of Molecular Neurology, Tokyo University of Pharmacy and Life Sciences, Hachioji, Tokyo 192-0392, Japan; you_gene@icloud.com (N.M.); miyamoto-y@ncchd.go.jp (Y.M.); ucqovp8er@gmail.com (K.H.); 2 Laboratory of Molecular Pharmacology, National Research Institute for Child Health and Development, Setagaya, Tokyo 157-8535, Japan; 3 Laboratory of Cell Signaling, Tokyo University of Pharmacy and Life Sciences, Hachioji, Tokyo 192-0392, Japan; aito@toyaku.ac.jp; 4 Laboratory of Oncology, Tokyo University of Pharmacy and Life Sciences, Hachioji, Tokyo 192-0392, Japan; hharada@toyaku.ac.jp; 5 Tsumura Research Laboratories, Tsumura & Co., Inashiki, Ibaraki 200-1192, Japan; ooizumi_hiroaki@mail.tsumura.co.jp (H.O.); oobuchi_katsuya@mail.tsumura.co.jp (K.O.); mizoguchi_kazushige@mail.tsumura.co.jp (K.M.)

**Keywords:** VPS11, HLD12, p70S6K, protein aggregate, oligodendroglial cell, morphological differentiation

## Abstract

Myelin sheaths created by oligodendroglial cells encase neuronal axons to achieve saltatory conduction and protect axons. Pelizaeus-Merzbacher disease (PMD) is a prototypic, hereditary demyelinating oligodendroglial disease of the central nervous system (CNS), and is currently known as hypomyelinating leukodystrophy 1 (HLD1). HLD12 is an autosomal recessive disorder responsible for the gene that encodes vacuolar protein sorting-associated protein 11 homolog (VPS11). VPS11 is a member of the molecular group controlling the early endosome antigen 1 (EEA1)- and Rab7-positive vesicle-mediated protein trafficking to the lysosomal compartments. Herein, we show that the HLD12-associated Cys846-to-Gly (C846G) mutation of VPS11 leads to its aggregate formation with downregulated signaling through 70 kDa S6 protein kinase (p70S6K) in the oligodendroglial cell line FBD-102b as the model. In contrast, wild-type proteins are localized in both EEA1- and Rab7-positive vesicles. Cells harboring the C846G mutant constructs decrease differentiated phenotypes with web-like structures following differentiation, whereas parental cells exhibit them suitably. It is of note that we identify PP1C and PP2A as the protein phosphatases for phosphorylated Thr-389 of p70S6K essential for kinase activation in cells. The respective knockdown experiments or inhibitor treatment stimulates phosphorylation of p70S6K and ameliorates the inhibition of morphological differentiation, as well as the formation of protein aggregates. These results indicate that inhibition of p70S6K phosphatases PP1C and PP2A improves the defective morphological differentiation associated with HLD12 mutation, thereby hinting at amelioration based on a possible molecular and cellular pathological mechanism underlying HLD12.

## 1. Introduction

In the central nervous system (CNS), oligodendrocytes (or oligodendroglial cells) wrap multiple layers of myelin sheaths around neuronal axons. Myelin sheaths are derived from differentiated plasma membranes. They often grow to more than 100 times larger than the collective surface area of the premyelinating plasma membranes of oligodendroglial cells [1,2,3,4]. Myelin sheaths play an indispensable role in the propagation of saltatory conduction. They also protect axons from physical and physiological stresses [1,2,3,4].

Hypomyelinating leukodystrophies (HLDs) are a group of recently classified hereditary neuropathies, primarily for oligodendroglial cells, that affect one out of every 200,000 to 500,000 people. Pelizaeus-Merzbacher disease (PMD) is a prototypic HLD, and is now named HLD1. The primary reason for myelination defects is incomplete or negligible oligodendroglial differentiation [5,6]. Defective myelination results in myelin dysfunction, complete demyelination, and, in turn, a loss of myelinated axons [5,6]. Thus, HLDs are considered severe neuropathies. HLD1 is associated with alternations of the *plp1* gene. The gene product is the major myelin structural, tetraspan-type membrane protein [7,8]. HLD2 is responsible for the *gjc2* (also called *gja12*) gene. It encodes the tetraspan membrane protein, which constitutes the gap junction [9]. These gene products are required for generating multiple layers of myelin sheaths and for maintaining them.

Recent advanced nucleotide sequencing technologies have enabled the identification of unexpected types of genes responsible for HLDs. The gene responsible for autosomal recessive HLD12 encodes vacuolar protein sorting-associated protein 11 homolog (VPS11) (OMIN ID 616683). VPS11 belongs to a family member of the vacuolar protein sorting-associated proteins involved in vesicle transport to vacuoles. In the network between the trans-Golgi and plasma membranes, the class C core vacuole/endosome tethering (CORVET) molecular complex mediates the accessing of vesicles to early endosomes, whereas the homotypic fusion and vacuole protein sorting (HOPS) complex mediates the fusion of late endosomes with lysosomes [10]. VPS11 is a common subunit of both CORVET and HOPS, and is localized in vesicles positive for early and late endosome markers. The Cys846-to-Gly (C846G) mutation in human VPS11 is genetically associated with HLD12 [11,12,13]. While expression levels of VPS11 proteins are downregulated by the C846G mutation in patient tissues, mutated VPS11 proteins are still expressed in them. It remains to be investigated whether and how the C846G mutant proteins of VPS11 have cell biological and biochemical effects in mammalian cells.

Here, we describe that the C846G mutant proteins of VPS11 form aggregates, with decreased signaling through 70kDa S6 protein kinases (p70S6K), thereby inhibiting morphological differentiation in mouse oligodendroglial FBD-102b cells as the model. Phosphorylated, activated p70S6K, acting through the mammalian target of rapamycin (mTOR) signaling, is known to promote oligodendroglial cell differentiation and myelination [14,15,16]. Furthermore, we identify PP1C and PP2A as serine and threonine protein phosphatases for p70S6K in cells. Their inhibition not only increases the phosphorylation of p70S6K, but also impairs the C846G mutant protein-associated defective morphological differentiation and aggregate formation. These results allow us to consider amelioration based on a possible pathological molecular and cellular mechanism triggered by HLD12-associated VPS11 mutant proteins.

## 2. Materials and Methods

### 2.1. Primary Antibodies

The following antibodies were purchased: mouse monoclonal antibody against a KDEL-containing peptide of the ER-resident Glucose-regulated protein (GRP78) (Cat. No. M181-3; immunofluorescence [IF], 1/200), anti-rabbit polyclonal early endosome antigen 1 (EEA1) (Cat. No. PM062; IF, 1/400), mouse monoclonal anti-actin (Cat. No. M177-3; immunoblotting [IB], 1/20,000), mouse monoclonal anti-DDDDK antigen (also called FLAG antigen, Cat. No. M185-3; IB, 1/20,000), and mouse monoclonal anti-GFP (Cat. No. M048-3; IB, 1/1,000) from MBL (Aichi, Japan); mouse monoclonal anti-lysosomal-associated membrane protein 1 (LAMP1) (Cat. No. ab25630; IF, 1/100), rabbit polyclonal anti-(pThr389) p70S6K/RPS6KB1, which recognizes a phosphorylated, activated state of p70S6K (Cat. No. ab2571; IB, 1/500), rabbit monoclonal anti-p70S6K (Cat. No. ab325291; IB, 1/2,500), rabbit monoclonal anti-(pSer240/244) S6 protein/RPS6, which recognizes a phosphorylated state by p70S6K (Cat. No. ab215214; IB, 1/10,000, and IF, 1/100), rabbit polyclonal anti-S6 protein/RPS6 (Cat. No. ab70227; IB, 1/500), rabbit monoclonal anti-(pThr37) 4E-BP1/eIF4E-BP1, which recognizes a phosphorylated state by mTOR kinase (Cat. No. ab75767; IB, 1/2,500; and IF, 1/200), rabbit monoclonal anti-4E-BP1 (Cat. No. ab32024; IB, 1/5000); rabbit monoclonal anti-ubiquitin (Cat. No. ab7254; IF, 1/300), and mouse monoclonal anti-vimentin (Cat. No. ab8069; IF, 1/200) from Abcam (Bristol, UK); mouse monoclonal anti-Golgi matrix protein of 130 kDa (GM130) (Cat. No. 610822; IF, 1/200) from BD Biosciences (Franklin Lakes, NJ, USA); rabbit polyclonal late endosomal Rab7 (Cat. No. 9367S; IF, 1/100) from Cell Signaling Technology (Danvers, MA, USA); and rabbit polyclonal proteolipid protein 1 (PLP1) (Cat. No. HPA004128; IB, 1/500) from Atlas Antibodies (Bromma, Sweden).

### 2.2. Inhibitors

Okadaic acid, an inhibitor for PP1 and PPP2A, and Hygromycin B, an antibiotic compound, were purchased from Nacalai Tesque (Kyoto, Japan).

### 2.3. Plasmid Constructions

The plasmid encoding the human full-length vacuolar protein sorting-associated protein 11 homolog (VPS11, GenBank Acc. No. NM_021729), tagged with *A. victoria* green fluorescence protein GFP-Spark at the C-terminus, was purchased from Sino Biological, Inc. (Wayne, PA, USA). The Cys846-to-Gly (C846G; 2536T-to-G in the nucleotide level) mutation was produced from the plasmid encoding VPS11 (OMIN ID 616683) as the template using a site-directed mutagenesis kit (Toyobo Life Science Department, Osaka, Japan), with two specific primers (Table 1), in accordance with the manufacturer’s instructions. Human full-length serine and threonine phosphatases (a catalytic subunit of the heteromultimeric protein complex or a single phosphatase protein) were amplified from SuperScript III reverse transcriptase (Thermo Fisher Scientific, Waltham, MA, USA)-mediated human brain cDNA (human RNA origin from Nippon Gene Co. Ltd., Tokyo, Japan) using Gflex DNA polymerase (Takara Bio, Shiga, Japan), in accordance with the manufacturer’s instructions, with the specific primer pairs (Table 1) of PPP1CA coding region (GenBank Acc. No. NM_002708); PPP1CC plus 3’-non-coding region (NM_002710), PPP2CA coding region (NM_002715), PPP2CB coding region (NM_001009552), PPP3CA coding region [NM_000944], PPP4C coding region [NM_001303503], PPP6C coding region (NM_001123355), PPM1B coding region (NM_002706), and PPM1G coding region (NM_177983). They were ligated into the mammalian GFP-expressing pEGFP-C1. The plasmid encoding rat p70S6K with FLAG-tag at the N-terminus was kindly provided by Dr. T. Torii (Doshisha University, Kyoto, Japan). All DNA sequences were confirmed by sequencing (Fasmac, Kanagawa, Japan).

### 2.4. RT-PCR and PCR Primers

The cDNAs were prepared from Isogen (Nippon Gene, Tokyo, Japan)-extracted total RNA with a PrimeScript RT Master Mix kit (Takara Bio), in accordance with the manufacturer’s instructions. PCR amplification was performed using ExTaq DNA polymerase (Takara Bio) with 26 to 30 cycles, each consisting of a denaturation reaction, an annealing one depending on primer annealing temperatures, and an extension one. The primers used for mouse myelin proteolipid protein 1 (PLP1), mouse myelin basic protein (MBP), mouse PPP1CC, mouse PPP2CA, and human and rodent VPS11 are described in Table 1. All primers were purchased from Fasmac.

### 2.5. Cell Culture, Differentiation, Plasmid Transfection, and Stable Clone Isolation

African green monkey kidney epithelial cell-like COS-7 cells were cultured on cell culture dishes (Greiner, Oberösterreich, Germany) in Dulbecco’s Modified Eagle Medium (DMEM, Thermo Fisher Scientific) containing 10% heat-inactivated FBS (GE Healthcare, Chicago, IL, USA) and PenStrep (Thermo Fisher Scientific) in 5% CO_2_ at 37 °C. COS-7 cells were purchased from JCRB Cell Bank (Osaka, Japan).

Mouse brain oligodendroglial FBD-102b cells were cultured on cell culture dishes in DMEM/Nutrient Mixture F-12 (Thermo Fisher Scientific) containing 10% heat-inactivated FBS and PenStrep in 5% CO_2_ at 37 °C. To induce differentiation, FBD-102b cells were cultured on cell culture dishes (Greiner) with advanced TC polymer modification in culture medium without FBS in 5% CO_2_ at 37°C for 5 days. Cells with large web-like membrane structures along multiple processes from cell bodies were considered to be differentiated ones. Differentiation was accomplished with expression of PLP1. FBD-102b cells were kindly provided by Dr. Y. Tomo-oka (Tokyo University of Science, Chiba, Japan).

Cells were transfected with the respective plasmids using a ScreenFect A or ScreenFect A Plus transfection kit (FujiFilm, Tokyo, Japan), in accordance with the manufacturer’s instructions. The medium was replaced 4 h after transfection and was generally used for experiments 48 h after transfection. Attached, trypan-blue (Nacalai Tesque)-incorporating cells were less than 5% in each experiment.

For the collection of FBD-102b cells stably harboring the C864G mutant constructs of VPS11, cells were transfected with plasmid encoding the C864G mutant in a 3.5 cm cell culture dish. Growth medium containing 200 g/mL Hygromycin B was changed every 2 or 3 days. After more than 14 days, Hygromycin B-resistant clones were collected and further cultured for an additional 14 days to be compared to the phenotypes of their parental cells.

### 2.6. siRNA Transfection and Target Sequences

Cells were transfected with the respective siRNAs using a ScreenFect siRNA transfection kit (FujiFilm), in accordance with the manufacturer’s instructions. The medium was replaced 4 h after transfection and was generally used for experiments 48 h after transfection. The siRNA target sequences for mouse PPP1CC, mouse PPP2CA, and control luciferase with no significant homology to any mammalian gene sequences were described in Table 2. Attached, trypan incorporating cells were less than 5% in each experiment.

### 2.7. Fluorescence Images

Cells on a coverslip were fixed with 4% paraformaldehyde (Nacalai Tesque) or 100% cold methanol (Nacalai Tesque). Cells were blocked with Blocking One reagent (Nacalai Tesque) and incubated first with primary antibodies and then with Alexa Fluor-conjugated secondary antibodies (Thermo Fisher Scientific). The coverslips on the slide glass were mounted with Vectashield reagent (Vector Laboratories, Burlingame, CA, USA). The TIFF images were collected with a microscope system equipped with a laser-scanning Fluoview apparatus (FV1000D or FV1200, Olympus, Tokyo, Japan) using Fluoview software (Olympus). The resulting colored images were analyzed in Fiji software version Java 8 (NIH, MD, USA; URL: https://imagej.net/Fiji) for line plots. Images in figures are representative of experimental results.

### 2.8. Polyacrylamide Gel Electrophoresis and Immunoblotting

Cells were lysed in lysis buffer (50 mM HEPES-NaOH, pH 7.5, 150 mM NaCl, 5 mM MgCl_2_, 1 mM phenylmethane sulfonylfluoride, 1 μg/mL leupeptin, 1 mM EDTA, 1 mM Na_3_VO_4_, 10 mM NaF, and 0.5% NP-40) and were prepared as described [17,18]. The samples were separated on pre-made sodium dodecyl sulfate-polyacrylamide gels (Nacalai Tesque). The electrophoretically separated proteins were transferred to a PVDF membrane (Merck Millipore, Darmstadt, Germany), blocked with Blocking One reagent, and immunoblotted using primary antibodies, followed by peroxidase-conjugated secondary antibodies (MBL). The bound antibodies were detected by X-ray film exposure using ImmunoStar Zeta reagent (FujiFilm). The films were captured as TIFF image files using a GT-X770 scanner (Epson, Nagano, Japan) and its driver software (Epson). The band pixels were measured in the segment analysis mode using UN-SCAN-IT software version 6 (Orem, UT, USA; URL: https://www.silkscientific.com/gel-analysis.htm). The pixel values of the protein bands were described as percentages and were compared to the control values. Images in figures are representative of experimental results.

### 2.9. Transcriptome Analysis

We have previously registered data for primary rat oligodendrocyte precursor cell mRNAs (GEO Acc. No. GSE114957). Briefly, total RNA was labelled with Cy5 using an Amino Allyl MessageAMP II aRNA Amplification kit (Thermo Fisher Scientific). A 3D-Gene chip (rat oligo chip 20k;) was used for microarray analysis by Toray 3D-Gene chip service (Toray). The signals, which were hybridized to Cy5-labelled aRNA pools, were obtained using a Toray 3D-Gene scanner (Toray). The detected signal values for the respective genes were normalized according to the global normalization method and were processed by Kamakura-Techno 3D-Gene analytical service (Kanagawa, Japan). The median value of the detected signal intensities was adjusted to 25 (Table 3).

### 2.10. Statistical Analysis

Values are means ± SD from separate experiments. Intergroup comparisons were performed by unpaired studenst’ t test using Microsoft Excel software versions 2011 and 2018 (Redmond, WA, USA; URL: https://www.microsoft.com). For more than three samples, one way analysis of variance (ANOVA) was followed by a Fisher’s protected least significant difference test as a post hoc comparison using AnalystSoft StatPlus software versions 5 and 6 (Walnut, CA, USA; URL: https://www.analystsoft.com/). Differences were considered significant when *p* < 0.05.

### 2.11. Ethics Statement

Gene recombination techniques were performed in accordance with a protocol approved by both the Tokyo University of Pharmacy and Life Sciences Gene and Animal Care Committees (Approval No. L20-04 and L20-05, 1 April 2020).

## 3. Results

### 3.1. The C846G Mutation Renders VPS11 Proteins to Form Aggresomes

**Figure 1:**
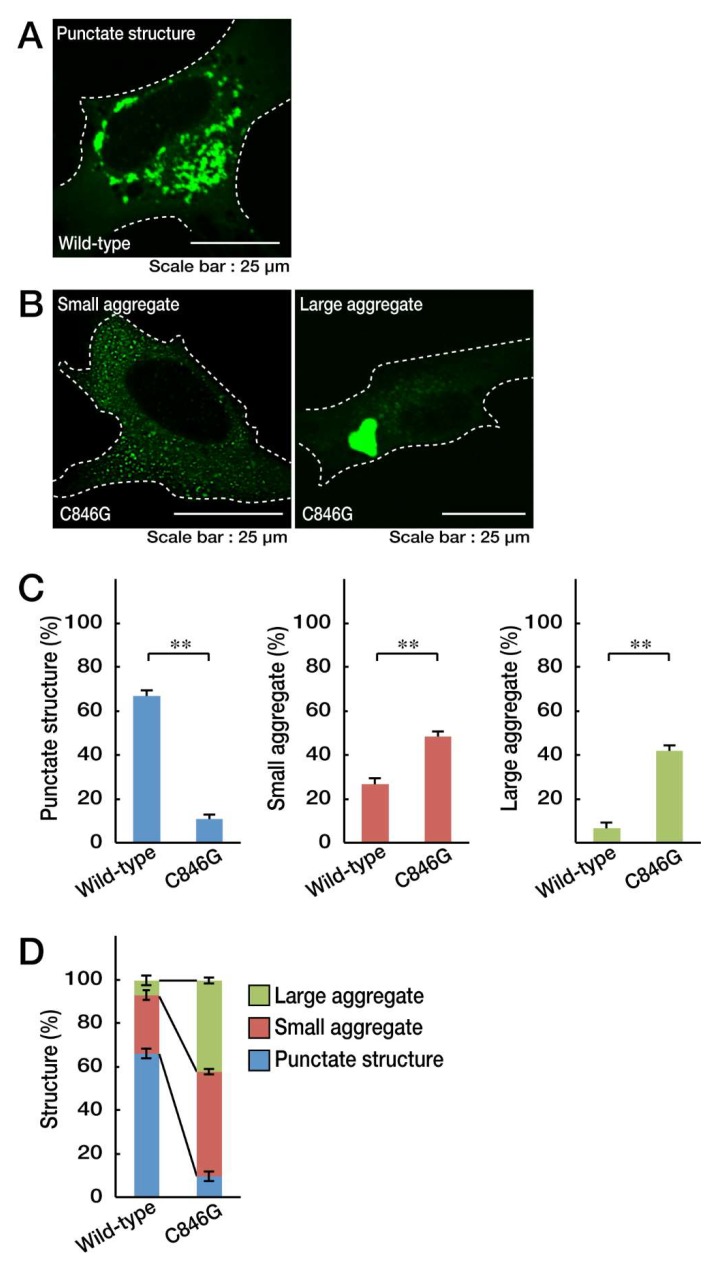
The Cys846-to-Gly (C846G) mutant proteins of vacuolar protein sorting-associated protein 11 homolog (VPS11) are present in small aggregates and large aggregates. **A**. FBD-102b cells were transfected with the plasmid encoding wild-type VPS11 with a GFP tag and were obtained as representative fluorescence images of punctate structures (green). **B**. Cells were transfected with the plasmid encoding the C846G mutant of VPS11 and were obtained as representative fluorescence images of small aggregates and large aggregates. **C**. The graph on the left shows the percentages of cells containing punctate structures (**, *p* < 0.01 in Student’s *t*-test; *n* = 3 fields [total 240 cells]). The graphs in the middle and on the right show the percentages of cells containing small aggregates and large aggregates (**, *p* < 0.01 in Student’s *t*-test; *n* = 3 fields [total 240 cells]). **D**. The percentages of cells with the respective structures are also shown in a graph.

To explore whether the localization of the C846G mutant proteins of VPS11 in cells differs from that of wild-type proteins, we transfected the plasmid encoding GFP-tagged human VPS11 or the C846G mutant into oligodendroglial cell line FBD-102b. Wild-type VPS11 proteins were distributed in punctate structures typical of transporting transport vehicles throughout the cytoplasm (Figure 1A,C,D). In contrast, mutant proteins were present in small- or micro-aggregate (pre-aggresome-like) as well as in large-aggregate (aggresome-like) structures (Figure 1B–D).

**Figure 2:**
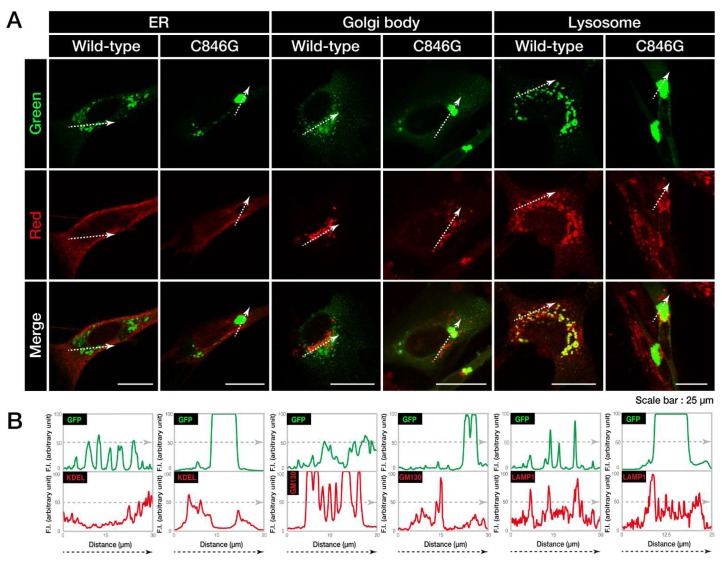
The C846G mutant proteins of VPS11 are not present in lysosomes. **A**. FBD-102b cells were transfected with the plasmid encoding wild-type or C846G VPS11 (green). Transfected cells were stained with the respective antibodies against the endoplasmic reticulum (ER), Golgi body, and lysosome markers KDEL, Golgi matrix protein of 130 kDa (GM130), and lysosomal-associated membrane protein 1 (LAMP1) (red). Merged images with the dotted arrows are also shown in the bottom panels. **B**. Fluorescence intensities (F.I., arbitrary unit) of green and red colors are shown along the dotted arrows in A.

First, to investigate where wild-type or C846G VPS11 proteins are localized in cells, we co-stained VPS11 proteins with the respective antibodies against the endoplasmic reticulum (ER), Golgi body, and lysosome (Figure 2A). Wild-type VPS11 proteins were co-stained with neither the ER marker KDEL, nor the Golgi body marker Golgi matrix protein of 130 kDa (GM130). They were co-stained with the lysosome marker lysosomal-associated membrane protein 1 (LAMP1). Co-localization of wild-type VPS11 proteins and LAMP1 is also shown as similar plot profiles in the line plot (Figure 2B), which is consistent with the established results that VPS11 acts in lysosomes and organelles around lysosomes [10]. The C846G mutant proteins were co-stained with neither KDEL nor GM130. In addition, they were not co-stained with LAMP1. In contrast, wild-type VPS11 proteins were present in both early endosome marker early endosome antigen 1 (EEA1)- and late endosome marker Rab7-stained vesicles. On the other hand, the C846G mutant proteins were not in either vesicles (Figure S1), suggesting that mutant proteins are not present in early and late endosomes but in other organelles and/or as the other structures.

**Figure 3:**
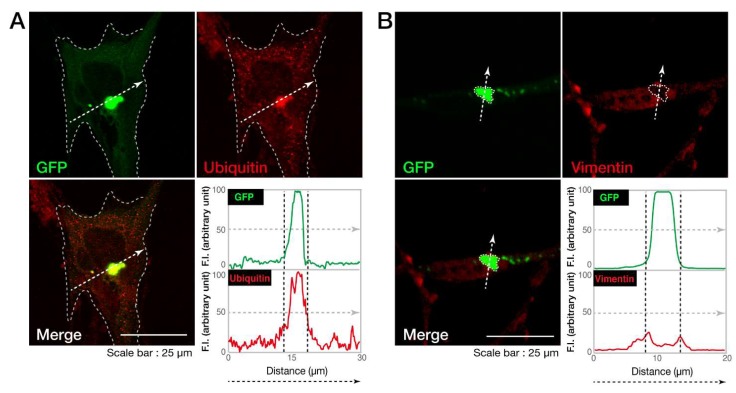
The C846G mutant proteins of VPS11 are positive with ubiquitin and surrounded by vimentin. **A** and **B**. FBD-102b cells were transfected with the plasmid encoding the C846G mutant of VPS11 (green). Transfected cells were stained with an antibody against ubiquitin or vimentin (red). Merged images with the dotted arrows are also shown in the bottom panels. Fluorescence intensities (F.I., arbitrary unit) of green and red are shown along the dotted arrows.

Next, we investigated whether the cellular aggregates of the C846G mutant proteins are possibly aggresomes. It is characterized that aggresomes are stained with an anti-ubiquitin antibody [19] and are surrounded by regions positive with an anti-vimentin antibody [20]. An anti-ubiquitin antibody detected the C846G mutant proteins (Figure 3A), which were also surrounded by vimentin marker (Figure 3B), indicating that the C846G mutation causes VPS11 proteins to form aggresomes.

### 3.2. Expression of the C846G Mutant Proteins in Cells Leads to Downregulation of p70S6K Signaling

**Figure 4:**
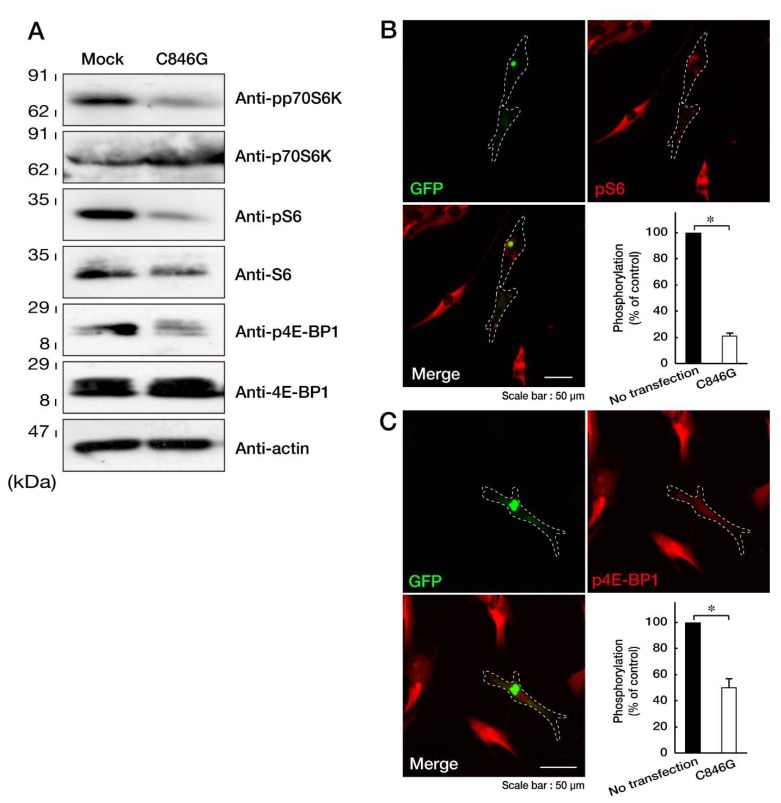
Expression of the C846G mutant proteins of VPS11 decreases phosphorylation of p70S6K, S6, and 4E-BP1 proteins. **A**. FBD-102b cells were transfected with mock or plasmid encoding the C846G mutant of VPS11, lysed, and immunoblotted with the respective antibodies against phosphorylated (p) p70S6K, S6, and 4E-BP1. Total p70S6K, S6, and 4E-BP1 and actin, as the internal control, are also shown. **B** and **C**. Cells were transfected with the C846G mutant (green) and stained with an antibody against phosphorylated (p) S6 or 4E-BP1 proteins (red). Right bottom panels show statistical data for the intensities of phosphorylated proteins in the C846G mutant or no transfected cells (*, *p* < 0.05 in Student’s *t*-test; *n* = 3 fields).

Since the formation of aggresomes is related to decreased p70S6K phosphorylation and activation through the mTOR signaling pathway [21,22], we investigated whether the expression of the C846G mutant proteins of VPS11 in cells decreases them. Transfection of the C846G mutant resulted in decreasing phosphorylated, activated p70S6K, whereas expression levels of p70S6K remained almost unchanged in the respective transfected cells (Figure 4A). Thr-389 of p70S6K is essential for its activation by phosphorylation [23,24]. Similar results were obtained in phosphorylation levels of S6 proteins as the substrate of p70S6K and 4E-BP1 proteins. 4E-BP1 is the direct substrate of mTOR kinase [23,24]. While expression levels of S6 proteins were changed by transfection with the C846G mutant, those of 4E-BP1 and internal control actin proteins were not changed by the transfection. It is likely that phosphorylated S6 proteins are stable for cellular proteolysis [25]. In immunofluorescence studies, we confirmed that cells expressing the C846G mutant but no transfected neighboring cells decreased phosphorylation levels of S6 and 4E-BP1 proteins (Figure 4B,C), revealing that the C846G mutation of VPS11 decreases p70S6K signaling.

### 3.3. Cells Stably Expressing the C846G Mutant Constructs Exhibit Undifferentiated Phenotypes

**Figure 5:**
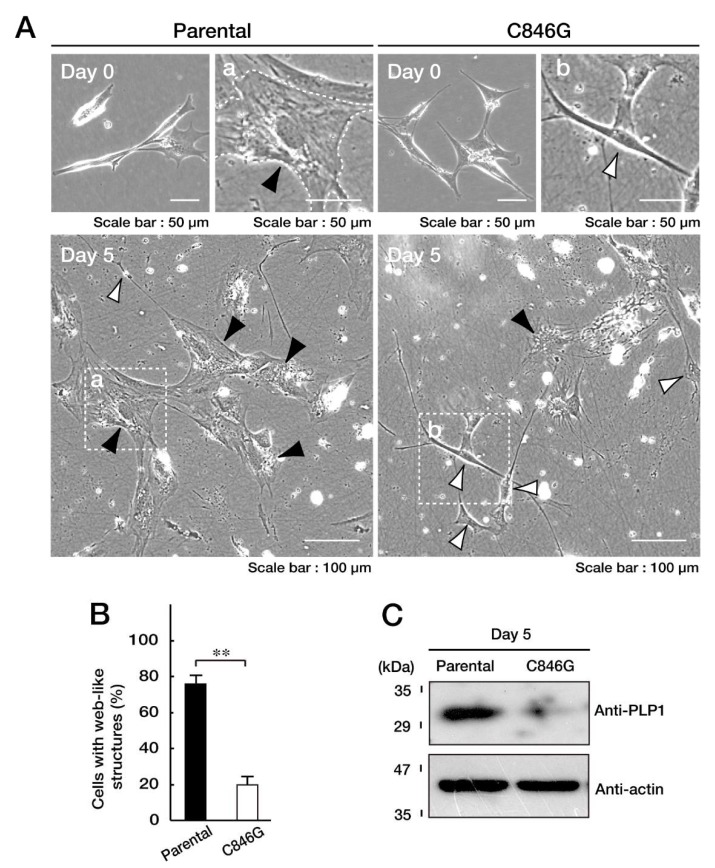
Cells harboring the C846G mutant constructs of VPS11 exhibit undifferentiated phenotypes with decreased myelin marker protein myelin proteolipid protein 1 (PLP1) expression. **A** and **B**. We allowed FBD-102b cells stably harboring the C846G mutant constructs or parental cells to differentiate for 0 or 5 days. Differentiated cells, ones with web-like structures, are statistically shown (**, *p* < 0.01 in Student’s *t*-test; *n* = 4 fields). Fields a and b in the bottom panels are also indicated as large images in the upper panels. Black and white arrowheads in the images indicate representative differentiated and undifferentiated cells, respectively. **C**. At day 5 following the induction of differentiation, cells were lysed and immunoblotted with an anti-PLP1 or actin antibody.

Although we tried to isolate the single cell-derived colonies following genome editing in the FBD-102b cells, we achieved neither colony isolation nor genome editing. Thus, we collected FBD-102b cells stably harboring the C846G mutant constructs and used them for experiments in cell differentiation (Figure S2). Cells were allowed to differentiate for 5 days. Before the induction of differentiation, FBD-102b cells exhibited spindle-like phenotypes with some processes. Following the induction of differentiation, FBD-102b cells exhibited phenotypes with large web-like structures along processes [26,27], and the transcription of myelin marker molecules such as myelin proteolipid protein 1 (PLP1) and myelin basic protein (MBP) (Figure S3), as seen in primary oligodendroglial cells [3,4,26,27]. C846G mutant clones failed to exhibit differentiated phenotypes, whereas parental cells did at a rate of approximately 70% (Figure 5A,B). Consistent with these results, stable clones exhibited decreased levels of markers such as PLP1 (Figure 5C). Together, it is thought that the C846G mutation of VPS11 leads to inhibition of FBD-102b cell differentiation.

### 3.4. PP1C and PP2A are p70S6K Phosphatases Whose Inhibition Impairs HLD12 Mutation-Associated Undifferentiated Phenotypes

**Figure 6:**
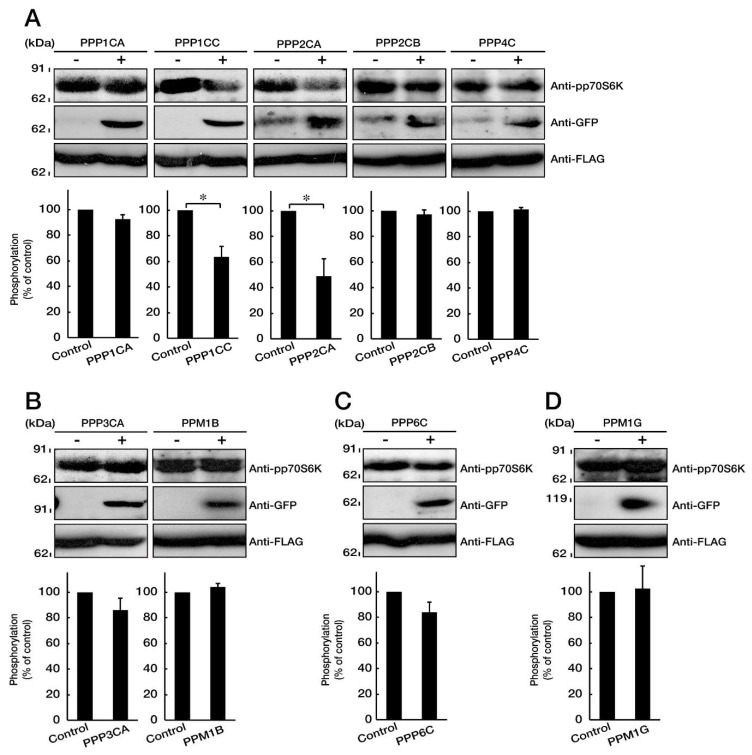
PP1C and PP2A are p70S6K phosphatases. **A**–**D**. COS-7 cells were transfected with mock (-) or the plasmid encoding the respective protein phosphatases (+, GFP-tag) with p70S6K (FLAG-tag). Cells were lysed and immunoblotted with an anti-phosphorylated (p) p70S6K antibody. Expression of GFP-tagged phosphatases and FLAG-tagged p70S6K is also shown. Statistical data for phosphorylated levels of p70S6K are shown in graphs (*, *p* < 0.05 in students’ *t*-test; *n* = 5 blots).

Signaling through p70S6K and its Thr-389 phosphorylation is required for oligodendroglial cell differentiation and myelination [15]. Since expression of the C846G mutant proteins decreases the phosphorylation of p70S6K in cells, we hypothesized that the C846G mutant-mediated undifferentiated phenotypes could be reversed by the increased phosphorylation of p70S6K, that is to say, by means of the inhibition of p70S6K phosphatases. We used rat oligodendroglial cell transcriptome data that we previously registered (GEO Acc. No. GSE114957). We entered the serine and threonine phosphatase term as the data query. We extracted PPP1CA, PPP1CC, PPP2CA, PPP2CB, PPP3CA, PPP4C, PPP6C, PPM1B, and PPM1G (Table 3). PPP1CA, PPP1CC, PPP2CA, PPP2CB, PPP3CA, PPP4C, and PPP6C are catalytic subunits of PP1A, PP1C, PP2A, PP2B, PP3A, PP4, and PP6, respectively. PPM1B and PPM1G are Mg^2+^- and Mn^2+^-dependent protein phosphatases. First, we transfected the plasmid encoding the respective GFP-tagged protein phosphatases with FLAG-tagged p70S6K into COS-7 cells. The p70S6K is phosphorylated in the growing conditions of cells [23,24,28]. Co-expression of PPP1CC or PPP2CA, but not of other respective phosphatases, decreased the phosphorylation levels of p70S6K (Figure 6A–D), suggesting that the inhibition of PPP1CC or PPP2CA may reverse the C846G mutant-mediated undifferentiated phenotypes in FBD-102b cells as well.

**Figure 7:**
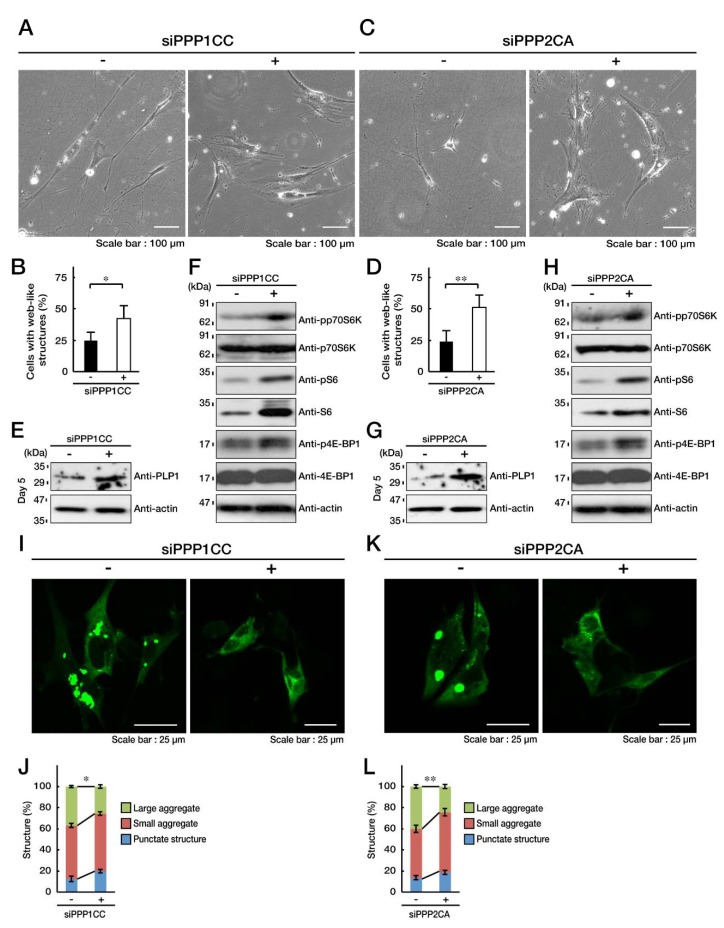
Knockdown of PP1C or PP2A impairs the C846G mutant-mediated undifferentiated phenotypes. **A** and **B**. FBD-102b cells harboring the C846G mutant constructs were transfected with siRNA for control luciferase (-) or PPP1CC (+), a catalytic subunit of PP1C, and were allowed to differentiate for 5 days. Cells with large web-like structures are statistically shown (*, *p* < 0.05 in Student’s *t*-test; *n* = 8 fields). **C** and **D**. Cells harboring the C846G mutant constructs were transfected with siRNA for control (-) or PPP2CA (+), a catalytic subunit of PP2A, and were allowed to differentiate. Cells with web-like structures are statistically shown (**, *p* < 0.01 in Student’s *t*-test; *n* = 8 fields). **E**–**H**. Following the induction of differentiation, expression levels of PLP1 and actin in control (-), PPP1CC (+), or PPP2CA (+) siRNA-transfected cells were confirmed by immunoblotting with the respective antibodies. Levels of phosphorylated (p) p70S6K, S6, and 4E-BP1 were confirmed by immunoblotting. Total p70S6K, S6, and 4E-BP1, and actin proteins are also shown. **I**–**L**. FBD-102b cells harboring the C846G mutant constructs (green) were transfected with siRNA for control (-), PPP1CC (+), or PPP2CA (+). Decreased large aggregates are statistically shown in the graph (**, *p* < 0.01 and *, *p* < 0.05 in one way ANOVA; *n* = 4 fields).

Next, we transfected each of four non-overlapping siRNAs for PPP1CC or PPP2CA into FBD-102b cells, and examined the effect of each siRNA on knockdown for PPP1CC or PPP2CA by RT-PCR (Figure S4). Effective for knockdown are four siRNAs for PPP1CC or three siRNAs for PPP2CA. We selected PPP1CC siRNA starting from the 78^th^ nucleotide or PPP2CA siRNA starting from the 109^th^ nucleotide, and employed them in the following experiments. Knockdown of PPP1CC impaired the C846G mutant-mediated undifferentiated phenotypes by approximately 50% (Figure 7A,B). Similarly, knockdown of PPP2CA impaired the undifferentiated phenotypes (Figure 7C,D). Their impairment was accomplished with expression of PLP1 (Figure 7E,G). Also, knockdown of PPP1CC or PPP2CA reversed phosphorylation levels of p70S6K as well as S6 and 4E-BP1 proteins (Figure 7F,H). In addition, knockdown of PPP1CC or PPP2CA impaired formation of large aggregates (Figure 7I,J; Figure 7K,L).

Together, PPP1CC or PPP2CA, a catalytic subunit of PP1C or PP2A, is characterized as a p70S6K phosphatase in cells, and inhibition ameliorates the C846G mutant-mediated undifferentiated phenotypes. PPP2CA has also been identified as the p70S6K phosphatase in both human prostate carcinoma cell line DU-145 and human hepatic cancer cell line HepG2 [29]. Phosphatases for p70S6K might vary depending on cell types, although we extracted two phosphatases of nine candidates by ectopic transfection experiments and determined that PP1C and PP2A are actually p70S6K phosphatases.

**Figure 8:**
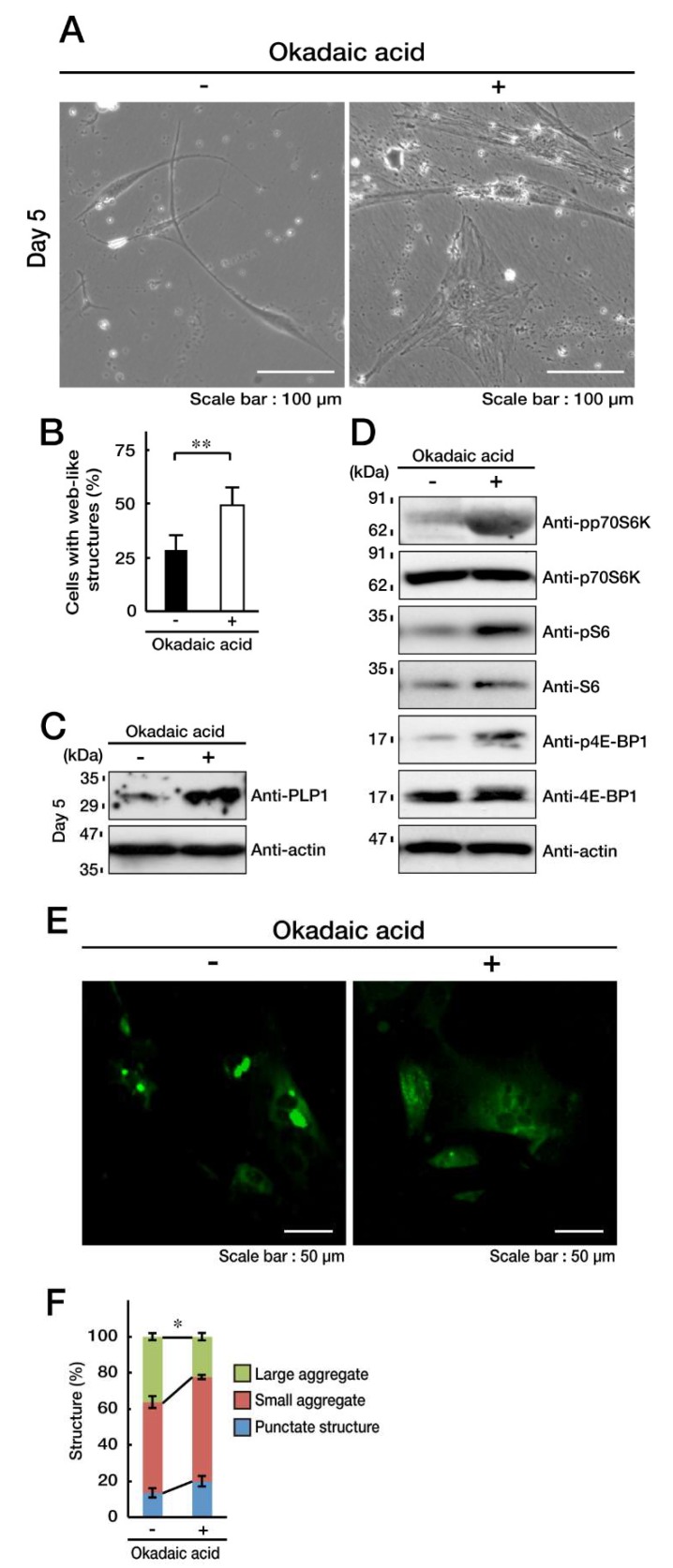
Okadaic acid, an inhibitor for PP1 and PP2A, impairs the C846G mutant-mediated undifferentiated phenotypes. **A** and **B**. FBD-102b cells harboring the C846G mutant constructs were treated with (+, inhibitor) or without (-, control vehicle) 1 nM Okadaic acid and were allowed to differentiate for 5 days. Cells with large web-like structures are statistically shown (**, *p* < 0.01 in Student’s *t*-test; *n* = 8 fields). **C**. Following the induction of differentiation, expression of PLP1 and actin in Okadaic acid-treated cells were confirmed by immunoblotting with the respective antibodies. **D**. Following the induction of differentiation, levels of phosphorylated (p) p70S6K, S6, and 4E-BP1 in Okadaic acid-treated cells were confirmed by immunoblotting. Total p70S6K, S6, 4E-BP1, and actin proteins are shown. **E** and **F**. Cells were allowed to differentiate in the presence (+) or absence (-) of Okadaic acid, and cells containing aggregate or punctate structures (green) were counted (*, *p* < 0.05 in one way ANOVA; *n* = 4 fields).

We next explored whether a chemical inhibitor has similar effects as the knockdown experiments of PPP1CC or PPP2CA. We treated cells stably harboring the C846G mutant constructs with Okadaic acid, which is a chemical inhibitor specific for both PP1 and PP2A [30]. Treatment with Okadaic acid impaired their undifferentiated phenotypes by approximately 50% (Figure 8A,B), and promoted PLP1 expression (Figure 8C). Also, it reversed the phosphorylation levels of p70S6K, as well as the S6 and 4E-BP1 proteins (Figure 8D). It is of note that Okadaic acid decreased the formation of large aggregates (Figure 8E,F).

Taken together with the results described above, the inhibition of PP1C or PP2A reverses p70S6K and its related signaling, impairing the C846G mutant-mediated undifferentiated phenotypes with aggregate formation.

## 4. Discussion

Defective oligodendroglial cell differentiation or myelin maintenance probably leads to a loss of myelinated axons following complete demyelination. The primary reason for a defect in myelination is complete or negligible oligodendroglial cell differentiation [5,6].

Studies on HLD1 indicate that incomplete oligodendroglial cell differentiation is due to unfolded protein response (UPR) specific for ER stress. HLD1 is associated with alternations of the *plp1* gene, whose products are the major myelin tetraspan membrane proteins. Various types of gene mutations lead to the accumulation of unfolded PLP1 proteins in the ER, triggering UPR [7,8]. UPR generally results in a change from being the response that triggers the cellular survival machine to one that commits the cell fate finally to the pathway to cell death. In the case of PLP1 mutations, they lead to the undifferentiated situation of cells or oligodendroglial cell death. Thus, the mutations are toxic-gain-of-function ones. Similar findings are observed in the cases of HLD2 and HLD5. The gene products responsible for HLD2 are membrane proteins constituting gap junctions, and their mutations result in unfolded GJC2 proteins accumulating in the ER [9]. The gene responsible for HLD5 encodes FAM126A (also called Hyccin or Drctnnb1a), which is a subunit of the cytoplasmic phosphatidylinositol 4-kinase enzyme. Also, the mutation of Leu53-to-Pro (L53P) leads to ER localization as unfolded, mutated FAM126A proteins, and triggers UPR, although it remains unclear how cytoplasmic proteins are transported in the ER by the mutation [31]. Differing from the cases of HLD1, HLD2, and HLD5, HLD12-associated VPS11 mutant proteins are not localized in the ER, but are present as VPS11 aggregates throughout the cytoplasm. Since the formation of aggresome-like structures is often responsible for decreasing cell homeostasis as a result of decreased mTOR signaling [21,22], the C846G mutation of VPS11 could be a toxic-gain-of-function one.

VPS11 is a component of a protein complex controlling vesicle docking and fusion [10]. The Cys-846 of VPS11 is positioned in a possible hinge region linking a region homologous with clathrin heavy chain arms (Pham ID: 00637) to a region homologous with the class C vacuolar protein sorting proteins (Pham ID: 12451). The C846G mutation may increase flexibility in the possible hinge region of VPS11, which plays a key role in the protein–protein interaction constituting the protein complex. It is possible that the C846G mutation results in forming an unstable protein complex containing mutated VPS11. In this context, the C846G mutation may also be a loss-of-function one, although a large part of mutated proteins are present as aggregates in cells.

The formation of protein aggresomes by missense mutations is a well-known phenomenon in Charcot-Marie-Tooth disease (CMTD), which is a demyelinating neuropathy of the peripheral nervous system [32,33]. While CMTD type IA, the major CMTD, is caused by various alterations in the *pmp22* gene, mutated *pmp22* gene products often accumulate as aggresomes or pre-aggresomes throughout the cytoplasm. Although the specific cytoplasmic signaling involved in aggresome formation has not been identified in demyelinating diseases, including CMTD, it is likely that the formation is correlated with decreased mTOR signaling [21,23]. The major outputs are p70S6K and S6 protein phosphorylation and 4E-BP1 phosphorylation [23,24]. The catalytic activity of p70S6K is regulated by multiple phosphorylation reactions located within the catalytic, linker, and pseudosubstrate domains [23,24]. Two major phosphorylation sites, Thr-229 and Thr-389, are important for the regulation of p70S6K. Phosphorylation of Thr-229 in the catalytic domain is contributed to by 3’-phosphoinositide-dependent kinase-1 (PDK1). In the linker domain, phosphorylation of Thr-389 by mTOR kinase is most critical for signal-dependent kinase regulation. Herein, we show that PP1C or PP2A acts as p70S6K Thr-389 phosphatases in cells. Decreased phosphorylation of Thr-389 by expression of the C846G mutant proteins of VPS11 is impaired by knockdown of PP1C or PP2A or inhibitor treatment. Knockdown of PP1C or PP2A also improves inhibitory morphological differentiation in cells harboring the C846G mutant constructs. Furthermore, aggregate formation is decreased through inhibition of their phosphatases. The elimination is likely related to increased p70S6K phosphorylation. It is of note that the inhibition of PP1C or PP2A impairs decreased phosphorylation of 4E-BP1 as well. 4E-BP1 is directly phosphorylated by mTOR kinase but not by p70S6K [28]. Thus, PP1A or PP2C may participate in dephosphorylation of some signaling molecules belonging to the mTOR pathway, although PP1C or PP2A actually contributes to dephosphorylation of p70S6K and the inhibition ameliorates HLD12-associated cellular phenotypes in FBD-102b cells.

In the present study, we demonstrate that the HLD12-associated C846G mutation of VPS11 causes mutant protein products to form aggregates, inhibiting morphological differentiation in FBD-102b cells. Cells harboring HLD12-associated VPS11 mutant constructs decrease signaling through p70S6K, which is critically involved in the regulation of oligodendroglial cell differentiation and myelination [14,15]. Inhibition of p70S6K phosphatase PP1C or PP2A by each siRNA knockdown or chemical inhibitor treatment impairs the inhibitory morphological differentiation with decreased formation of VPS11 aggregates. Further studies will enhance our understanding of not only how HLD12-associated VPS11 mutant proteins form aggregates to inhibit morphological differentiation, but also whether PP1C or PP2A dephosphorylates molecules other than p70S6K, possibly to impair the in vitro HLD12-associated cellular phenotypes. Elucidation of these detailed mechanisms may clarify the molecular and cellular relationship behind HLD12-associated phenotypes in vivo, as well as in vitro.

This study could provide some previously unknown potential therapeutic molecular targets for HLD12 and other HLDs. First, the finding that aggresome formation is associated with HLD12 may lead to potential aggresome-depleting chemicals [32,33] being valuable for cell pathological phenotypes of HLD12. Second, HLD12 and other HLDs may be rescued by inhibition of the p70S6K phosphatases PP1C and PP2A, since downregulation of molecules belonging to mTOR signaling pathway, including p70S6K, is often observed in these diseases [3,4,5,6]. Third, although Okadaic acid improves cell pathological phenotypes of HLD12, Okadaic acid is toxic for cells. It may be useful to synthesize a highly safe compound based on the structure of Okadaic acid. Also, it may be important to determine whether it has potential as a therapeutic chemical structure. In any case, studies in these lines can shed light on the development of drug-targeted medicine in HLDs and related diseases.

## Figures and Tables

**Table 1:** Oligonucleotide sequences for mutagenesis, human phosphatase isolation, and RT-PCR primers.

Names for Genes or Uses	5’ Primers	3’ Primers
the C846G mutagenesis of human VPS11	5’-ggctttgagagttactcggaaagtg-3’	5’-gtgttggtggaaggagtggc-3’
human PPP1CA coding region (GenBank Acc. No. NM_002708)	5’-ccgggatccatgtccgacagcgagaagctc-3’	5’-ccgggatccctatttcttggctttggcggaattg-3’
human PPP1CC coding region plus 3’-non-coding region (GenBank Acc. No. NM_002710)	5’-ccgggatccatggcggatttagataaactcaacatcgac-3’	5’-ccgggatcctcactcgtatagaacagtattgtttctataatttgaag-3’
human PPP2CA coding region (GenBank Acc. No. NM_002715)	5’-ccgggatccatggacgagaaggtgttcaccaag-3’	5’-ccgggatccttacaggaagtagtctggggtacgac-3’
human PPP2CB coding region (GenBank Acc. No. NM_001009552)	5’-ccgggatccatggacgacaaggcgttcac-3’	5’-ccgggatccttataggaagtagtctggggtgcg-3’
human PPP3CAcoding region (GenBank Acc. No. NM_000944)	5’-ccgggatccatgtccgagcccaaggcaattg-3’	5’-ccgggatcctcactgaatattgctgctattactgccattg-3’
human PPP4C coding region (GenBank Acc. No. NM_001303503)	5’-ccgggatccatggcggagatcagcgacc-3’	5’-ccgggatcctcacaggaagtagtcggccac-3’
human PPP6C coding region (GenBank Acc. No. NM_001123355)	5’-ccgggatccatggcgccgctagacctg-3’	5’-ccgggatcctcaaaggaaatatggcgttgtcgttctg-3’
human PPM1B coding region (GenBank Acc. No. NM_002706)	5’-ccgagatctatgggtgcatttttggataaacccaaaac-3’	5’-ccgagatcttcatattttttcaccactcatctttgtccctg-3’
human PPM1G coding region (GenBank Acc. No. NM_177983)	5’-ccgggatccatgggtgcctacctctcccag-3’	5’-ccgggatccctagtctcgcttggccttcttcttc-3’
mouse PLP1	5’-atgggcttgttagagtgttgtgctagatgtctg-3’	5’-gaacttggtgcctcggcccatgag-3’
mouse MBP	5’-atggcatcacagaagagaccctcac-3’	5’-cccttgaatcccttgtgagccg-3’
rodent actin	5’-atggatgacgatatcgctgcgctc-3’	5’-ctagaagcatttgcggtgcacgatg-3’
mouse PPP1CC	5’-atggcggatatcgacaaactcaacatcg-3’	5’-ctttgcttgctttgtgatcataccccgtg-3’
mouse PPP2CA	5’-atggacgagaagttgttcaccaaggag-3’	5’-caggaagtagtctggggtacgacgag-3’
human and rodent VPS11	5’-tctgaggagttcatccccatctttg-3’	5’-gccacacaggaagtggactgag-3’

**Table 2:** Target sequences for mouse and luciferase siRNAs.

Names	Target Sequences
78th nucleotide number of mouse PPP1CC	5’-aagaatgtccagctccaggag-3’
123th nucleotide number of mouse PPP1CC	5’-aagtctcgggagatcttcctc-3’
163th nucleotide number of mouse PPP1CC	5’-aacttgaagcaccactcaaga-3’
258th nucleotide number of mouse PPP1CC	5’-aactatttgtttctcggggac-3’
87th nucleotide number of mouse PPP2CA	5’-aagagcctctgcgagaaggct-3’
109th nucleotide number of mouse PPP2CA	5’-aagaaatcctgacaaaagaat-3’
139th nucleotide number of mouse PPP2CA	5’-aagaggttcgatgtccagtca-3’
312th nucleotide number of mouse PPP2CA	5’-aaggttcgttaccgagagcgc-3’
control luciferase	5’-aagccattctatcctctagag-3’

**Table 3:** Transcriptome analysis of serine and threonine phosphatases in oligodendroglial cells. Transcriptome analysis was performed using total RNA from primary rat oligodendrocyte precursor cells. Effective values are shown in yellow.

GenBank Acc. No.	Name	Sample 1	Sample 2	Sample 3	Average	S.D.	Coefficient of Variation
NM_031527.1	PPP1CA	1436	2355	2147	1979	481.743	0.243
NM_022498.1	PPP1CC	1457	1673	1356	1495	161.750	0.108
NM_017039.2	PPP2CA	770	1385	994	1049	311.223	0.297
NM_134359.1	PPP4C	279	745	391	472	242.878	0.515
NM_017040.1	PPP2CB	287	527	366	393	122.327	0.311
NM_147209.2	PPM1G	254	415	333	334	80.172	0.24
NM_133589.2	PPP6C	251	356	249	285	61.148	0.215
NM_033096.1	PPM1B	178	116	97	130	42.433	0.325
NM_017041.1	PPP3CA	133	124	73	110	32.030	0.291

## Supplementary Materials

Supplementary materials can be found at https://www.mdpi.com/2227-9059/8/4/89/s1.
