# Supplementary material for: PP1C and PP2A are p70S6K Phosphatases Whose Inhibition Ameliorates HLD12-Associated Inhibition of Oligodendroglial Cell Morphological Differentiation
Source: Biomedicines. 2020 Apr 16;8(4):89. doi: 10.3390/biomedicines8040089 (PMC7235839; doi:10.3390/biomedicines8040089)
Supplement: Supplementary file 1 [file biomedicines-08-00089-s001.pdf]

Supplemental figure legends

Figure S1. The C846G mutant proteins of VPS11 are not localized in EEA1- and Rab7-positive vesicles where wild-type VPS11 is localized. FBD-102b cells were transfected with the plasmid encoding wild-type or C846G VPS11 (green). Transfected cells were stained with an antibody against EEA1 or Rab7 (red). Merged images with the dotted arrows are also shown in the panels. Fluorescence intensities (F.I., arbitrary unit) of green and red colors along the dotted arrows are shown in the bottom panels.

Figure S2. Transcription of VPS11 in stable clones harboring the C846G mutant constructs of VPS11 was confirmed. Total RNA was extracted from FBD-102b cells harboring the C846G mutant constructs of VPS11 and parental cells. RT-PCR was performed using a common primer pair for human and mouse VPS11 as well as the internal control actin.

Figure S3. Stable clones harboring the C846G mutant constructs of VPS11 exhibit unchanged profiles for differentiation (myelin) markers following the induction of differentiation. Total RNA was extracted from FBD-102b cells harboring the C846G mutant constructs of VPS11 and parental cells. RT-PCR was performed using primer pairs specific for mouse myelin markers PLP1, MBP, and actin (**, $p < 0.01$ and *, $p < 0.05$ in Student's t-test; n = 3).

Figure S4. Effects of four non-overlapping siRNAs for PPP1CC or PPP2CA on knockdown were evaluated. Total RNA was extracted from FBD-102b cells transfected with the respective siRNAs for control luciferase, PPP1CC, or PPP2CA. RT-PCR was performed using primer pairs specific for PPP1CC or PPP2CA and actin. All siRNA target sequences except for siRNA starting from the 87th nucleotide number of PPP2CA were effective for knockdown.

Figure S1

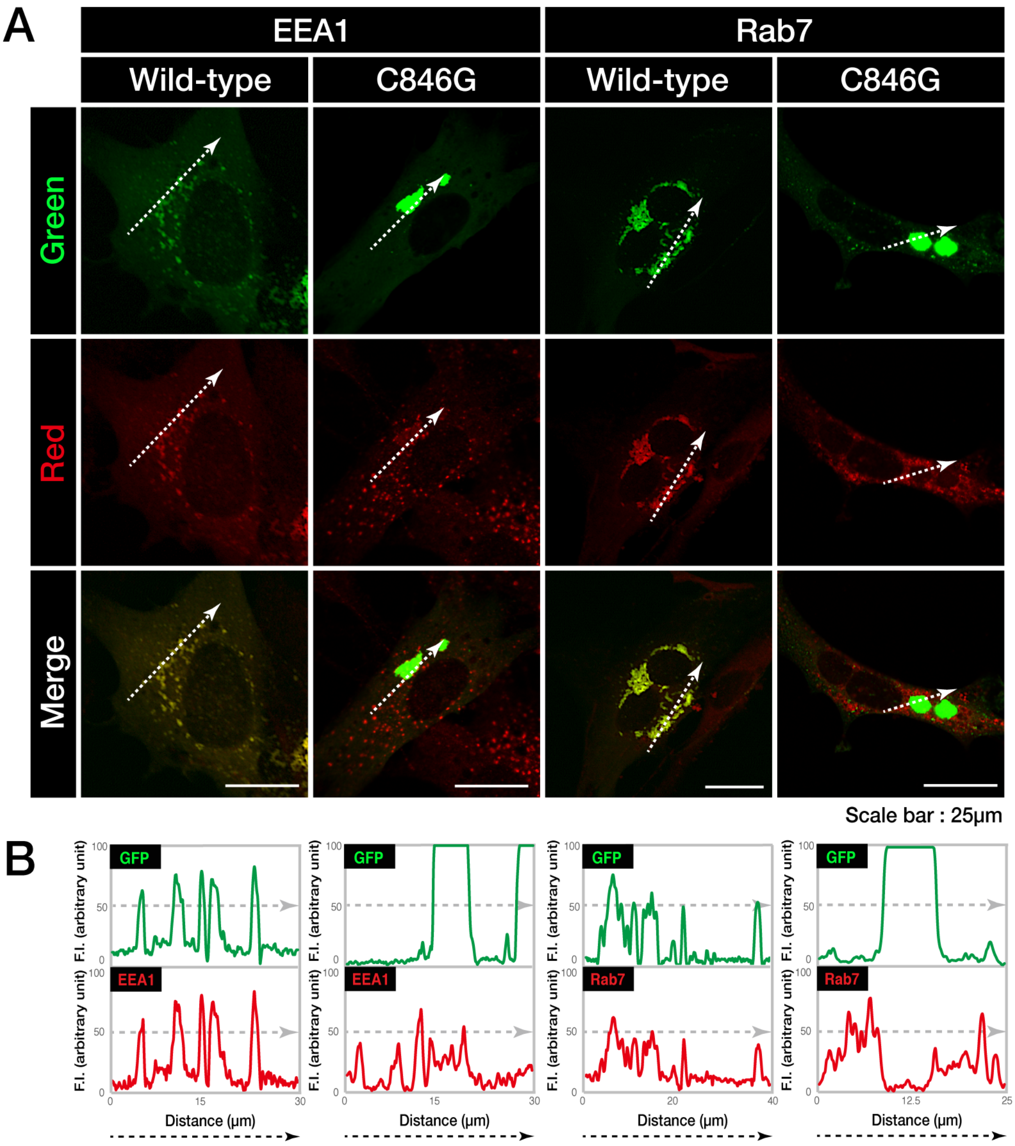
A
EEA1
Rab7
Wild-type
C846G
Wild-type
C846G
Green
Red
Merge
Scale bar : 25μm
B
GFP
EEA1
Rab7
F.I. (arbitrary unit)
Distance (μm)

Figure S2

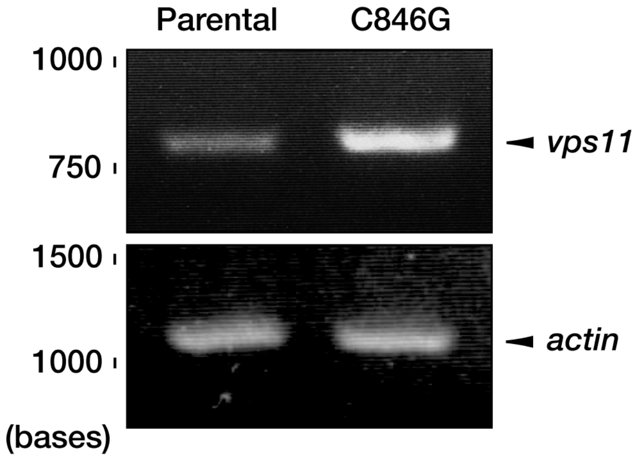

Figure S3

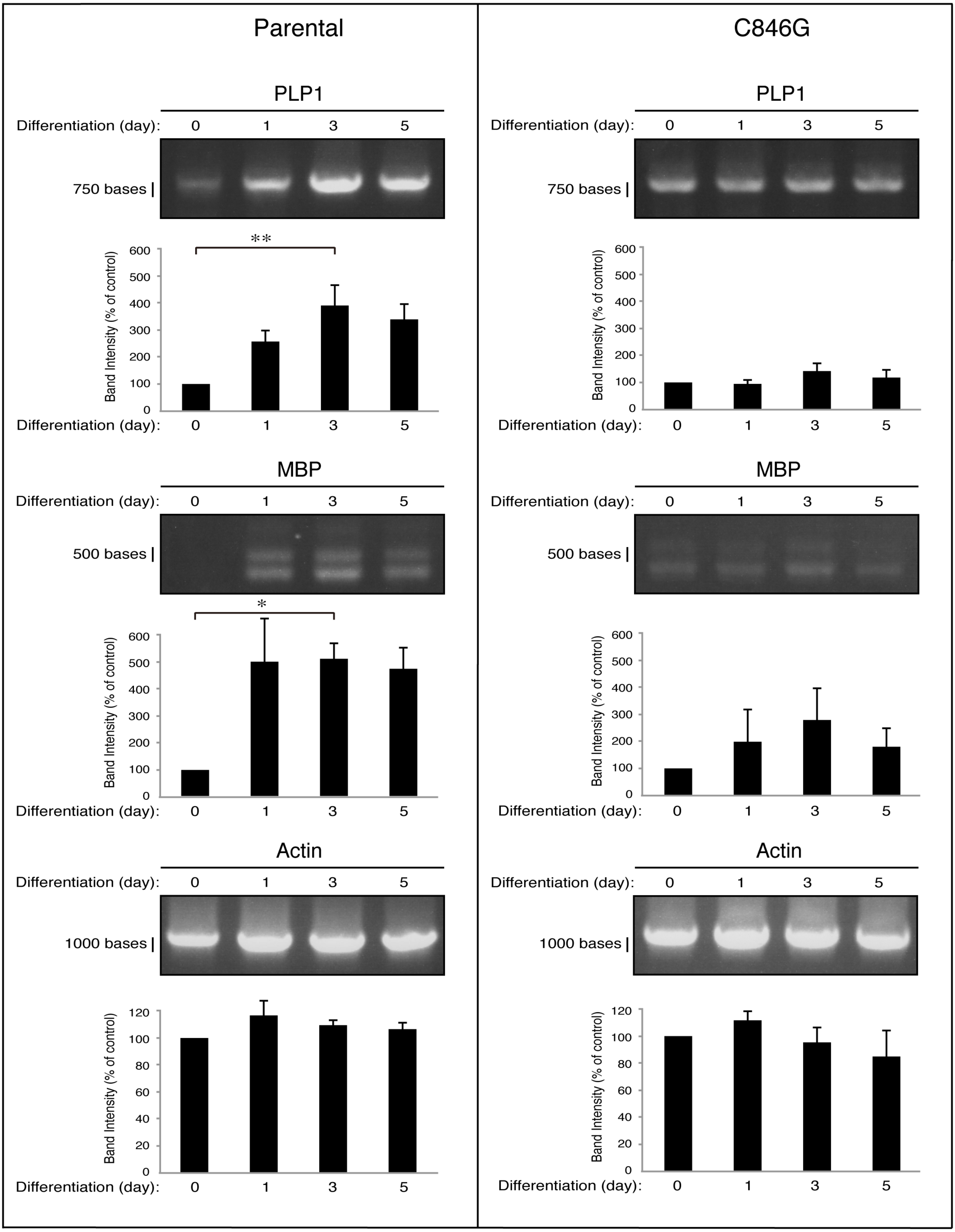

Parental
C846G
PLP1
MBP
Actin
Differentiation (day): 0 1 3 5
750 bases
500 bases
1000 bases
Band Intensity (% of control)
0
100
200
300
400
500
600
20
40
60
80
120
**
*

Figure S4

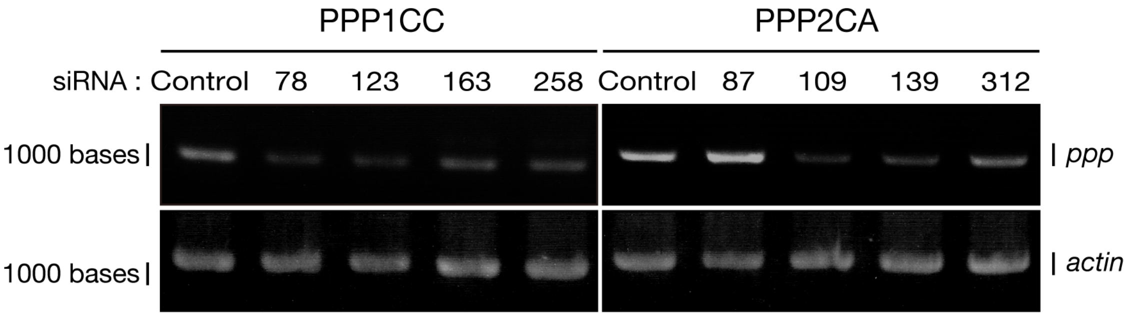
PPP1CC
PPP2CA
siRNA : Control 78 123 163 258 Control 87 109 139 312
1000 bases
1000 bases
ppp
actin
